# How Providing Cervical Cancer Screening Results via Cell Phone Affects Patient Follow-Up Rates in Western Kenya

**DOI:** 10.1200/JGO.18.00264

**Published:** 2019-06-27

**Authors:** Megan J. Huchko, Ibrahim Saduma, Cinthia Blat, Sandra Oketch, Elizabeth A. Bukusi

**Affiliations:** ^1^ Duke University, Durham, NC; ^2^ Kenya Medical Research Institute, Nairobi, Kenya; ^3^ University of California San Francisco, San Francisco, CA; ^4^ Aga Kahn University, Nairobi, Kenya

## Abstract

**Purpose:**

Human papillomavirus (HPV) testing is being more widely used in simplified cervical cancer screening protocols in low-resource settings. One challenge to successful implementation is the multiple visits necessary to provide results and follow-up. mHealth strategies may reduce visit burden by providing information through text message.

**Methods:**

As part of a cluster-randomized trial to compare HPV testing in clinics and community health campaigns in western Kenya, we carried out a mixed-methods study to assess women’s preferences and experiences with different strategies to receive their results. Women could opt to receive their HPV results via text message, cell phone call, home visit, or return clinic visit. We examined overall receipt of results, follow-up rates, and acceptability by notification method.

**Results:**

Among the 4,947 women who underwent HPV-based cervical cancer screening, 1,596 (32%) received results via text message, 1,181 (24%) via cell phone call, 1,563 (32%) via clinic visit, and 605 (12%) via home visit. Women opting for texts or calls were younger and had higher rates of prior cervical cancer screening, HIV testing, and modern contraceptive use (*P* < .001 for all). Home visits were associated with a significantly higher rate of treatment acquisition (45%) than texts (38%), cell phone calls (38%), and clinic visits (23%; *P* < .001). In a model controlling for age, prior screening, HIV testing, and contraceptive use, clinic visits remained significantly associated with decreased odds of treatment (adjusted odds ratio, 0.45; 95% CI, 0.29 to 0.69) compared with texts. Among treated women, there was no difference in time to treatment by notification method.

**Conclusion:**

Cell phone–based results notification strategies were preferred by women with greater health-seeking behavior; however, HPV-positive women who received results via home visit were more likely to pursue for treatment.

## INTRODUCTION

Cervical cancer remains an important public health issue in many low-resource countries, including Kenya, where the human papillomavirus (HPV) vaccine is not widely available and effective screening coverage is low.^1,2^ To address the infrastructural limitations in many low-resource settings, the WHO has developed guidelines for a simplified screen-and-treat strategy of HPV-based testing followed by cryotherapy for women who test positive.^3^ A screen-and-treat strategy with HPV is more effective than visual screening methods at reducing the risk of cervical intraepithelial neoplasia and cancer, provides a simple dichotomous test result, and allows for testing via self-collection.^3-5^ These characteristics facilitate screening outside clinic settings, potentially reaching a greater population.

**CONTEXT**

**Key Objective**
mHealth interventions are increasingly being adopted to bridge health care gaps in low-resource settings. We sought to understand the preferences of women in rural western Kenya around cell phone–based notification strategies for human papillomavirus test results and the impact of their notification choice on appropriate follow-up.
**Knowledge Generated**
Approximately two thirds of women had access to a cell phone and felt comfortable receiving results via call or text message. Cell phone preference was highly correlated with other health-seeking behaviors; however, women who received results by cell phone call or text message were not more likely to access treatment.
**Relevance**
These findings should prompt further investigation into cell phone use patterns among women in rural areas. Strategies for results notification and health messaging must ensure options for women without access to cell phones and provide content that is understandable and effective.

In real-world settings, however, the effectiveness of HPV-based screening programs is decreased by the need for multiple visits for screening, results, and indicated follow-up.^6^ Despite the increasing availability of low-cost HPV testing, there currently is no available point-of-care HPV test, making a same-day screen-and-treat strategy impossible. Even the most simplified strategies require two to three visits: screening, results notification, and, for HPV-positive women, treatment. Notification strategies must balance the need to provide adequate information about the implications of the results and the next steps for follow-up with the need to minimize clinic visits for women. In addition, results notification should be inexpensive and capable of reaching women in remote areas.

One strategy to reduce visit burden for both providers and patients is to provide results and follow-up information through a text message. Potential advantages of this mHealth strategy over follow-up through a clinic or home visit include cost, time, and privacy.^7^ Various strategies employing mHealth have been successfully implemented to support antenatal care and family planning in sub-Saharan Africa; however, relatively few programs or studies have evaluated mHealth in other reproductive health areas, such as cervical cancer screening.^8^ Although mHealth has filled gaps in health care access in certain settings, some studies have found that mHealth interventions might not reach women who have low health literacy and that many women, especially in rural areas, may be dependent on male partners for cell phone access, limiting their access to and comfort with text-based results.^9-11^

We sought to evaluate an approach that included two cell phone–based options (text messages and calls) to provide HPV results in rural Kenya, where cervical cancer rates are high and screening coverage is limited.^1,12^ Kenya has high rates of cell phone ownership,^13^ and mHealth strategies have been shown to improve postcircumcision care and adherence to prenatal and postpartum care among HIV-infected women.^14-16^ To our knowledge, little work has been done using mobile technology in cervical cancer prevention programs, which may have more potential for stigma or need for confidentiality compared with maternity care. We tested a cell phone–based results notification strategy as part of a larger trial to evaluate implementation strategies for HPV-based cervical cancer screening in rural western Kenya. We examined the prevalence of cell phone ownership, cell phone use, and willingness to receive cervical cancer screening results by cell phone, as well as participant characteristics associated with each method. We also analyzed the relationship between notification method, rate of follow-up, and time to treatment acquisition.

## METHODS

We carried out this mixed-methods study as part of a cluster-randomized trial comparing cervical cancer screening implementation strategies in Migori County, a rural area in western Kenya (ClinicalTrials.gov identifier: NCT2124252). The parent study enrolled women living in the area who were eligible for cervical cancer screening per Kenyan guidelines (age 25 to 65 years with an intact uterus and cervix). Twelve communities were assigned through a one-to-one randomization scheme to offer self-collected HPV testing either in community health campaigns (CHCs) held over a 2-week period, during which the entire population of eligible women in the community was targeted for screening (intervention arm), or in existing Kenyan government health facilities, where all eligible women presenting for outpatient services were targeted (control arm) throughout the 9-month study period.

HPV testing for both arms was performed using careHPV (QIAGEN, Germantown, MD) in a study-specific laboratory located within the Migori County Hospital. In the CHC arm, specimens were collected and brought to the laboratory on a daily basis. In the health facility arm, results were collected and returned to the clinic on a weekly basis. Results notification strategies and follow-up referrals were the same in both arms. Details about the study design and main outcomes are described in detail elsewhere.^17^

### Notification Strategies

**FIG 1:**
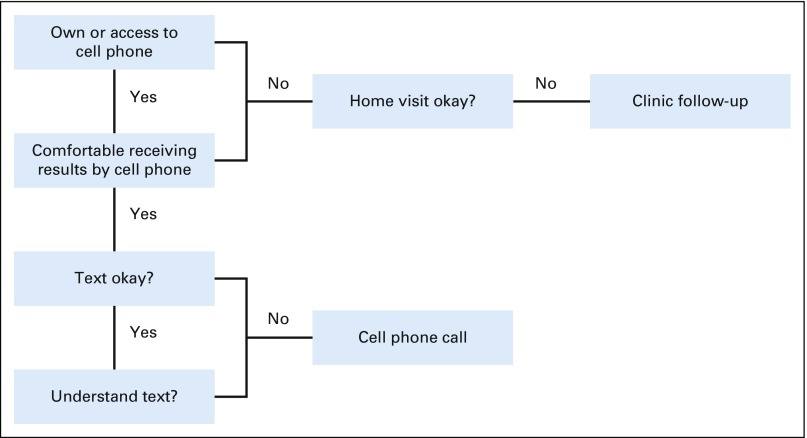
Strategy to determine the optimal method to provide results of HPV self-testing.

After enrollment, women who said they had their own cell phone or access to a cell phone with which they felt comfortable receiving results were offered the option of receiving their results via text message or call (Fig 1). The text message content was developed through focus group discussions, piloted before study launch (Table 1), and shown to participants at the time of screening to ensure understanding. Text messages were sent out in the participants’ preferred language (Kiswahili, Dhluou, or English) using Frontline SMS software (https://www.frontlinesms.com/), which indicated whether the message had been sent successfully or failed to reach the participant’s cell phone. Frontline SMS does not provide identity or message-read confirmation. Cell phone calls were made by trained study staff, who asked the participant to confirm her name, date of birth, and study participation before sharing results and follow-up information. Participants were able to ask any questions and provided with a number to text or call if they wanted clarification later.

**TABLE 1:**
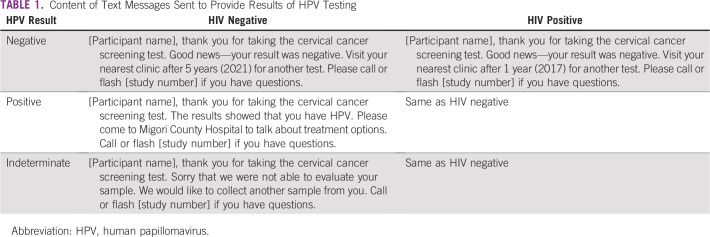
Content of Text Messages Sent to Provide Results of HPV Testing

Women who did not own or feel comfortable using a cell phone were offered the option of returning to the clinic where they were screened (for those in health facility arm) or their nearest clinic (for those in the CHC arm) or obtaining their results through a home visit from a community health volunteer (CHV) or a study assistant. For HPV-positive women who did not collect their results from the clinic within 4 weeks, follow-up via a home visit occurred. Participants were analyzed within the notification category chosen at the time of study enrollment.

The study tracked the process and success of results notification through an electronic database generated by Frontline SMS and paper-based logs entered into an Excel spreadsheet for the other notification methods. In the logs, study staff noted the screening results, notification method, number of attempts to reach the participant, whether and when notification was successful, and comments on why it was not. Research assistants attempted four cell phone calls or three home visits before determining that a participant was unable to be reached, and results were provided to their local clinic. For participants who opted for a clinic visit, lists of results were provided to the clinics on a weekly basis. When the participant returned to collect her results, CHVs at the clinic assigned to the study would indicate that the results were received and enter the date. For results confirmed to have been received but missing the date of receipt, the date that results were delivered to the clinic was used as the notification date. Paper-based data from these sources was entered into an electronic database approximately every 6 weeks.

### Treatment

Women who tested HPV positive were referred for evaluation for cryotherapy at Migori County Hospital. Before treatment, all women underwent a visual inspection with acetic acid to ensure that they did not have any contraindications to cryotherapy and did not need a more extensive procedure, such as a loop electrosurgical excision procedure, or referral for evaluation of invasive cancer. Treatment was provided by trained study nurses, who filled out a treatment information sheet before initiating therapy. These forms were collected by study staff and entered into tablets on a daily basis and were used to ascertain whether the participant successfully acquired treatment.

### Data Analysis

Data were queried for quality and completeness, summarized using descriptive statistics, and then analyzed for bivariate relationships using SAS software (SAS Institute, Cary, NC). Comparisons between notification groups were performed using χ^2^ or Fisher’s exact tests for categorical variables and Kruskal-Wallis test for continuous variables. A two-sided *P* value of < .05 was considered significant. We created two multivariable models using R software (version 3.3.3; https://www.r-project.org/) to better characterize the impact of the notification methods on timely treatment acquisition. Variables that were found to be significant in the bivariate analysis were included in a multivariable logistic regression to examine the relationship between notification type and treatment uptake among all HPV-positive women. A linear regression model was created to examine time to treatment among the subgroup of HPV-positive women who accessed treatment within the study period. For this analysis, we investigated notification success and treatment uptake within 4 months of screening date. Women who did not receive results or obtain treatment within that time continued to undergo intensified tracking by the research team; however, they were not included in the analysis.

### Ethical Approval

This study received ethical approval from the Kenya Medical Research Institute, Duke University institutional review board, and University of California San Francisco Committee for Human Subjects Research. All participants provided written consent at the time of screening.

## RESULTS

Between January and September 2016, 4,947 women underwent cervical cancer screening with self-collected HPV tests in clinics (n = 2,046) or CHCs (n = 2,898), and 1,047 (21%) tested HPV positive. Among the 376 HPV-positive women (36%) who accessed treatment, 337 (91%) did so within 3 months of receiving their results.^17^

At the time of screening, 3,570 women (72%) reported that they had their own cell phone or had access to one that they felt comfortable using (Table 2). Approximately one third of women opted to receive their results via text message (n = 1,596; 32%), and another third opted for a return clinic visit (n = 1,563; 32%). The remaining participants were split between cell phone call (n = 1,181; 24%) and home visit (n = 607; 12%). Among women opting for a home visit or return clinic visit, 136 (22%) and 658 (42%), respectively, reported access to a cell phone. There was a significant difference between the study arms in access to cell phones (CHC arm, 77% *v* health facility arm, 65%; *P* < .001) and in choice of notification method. In the CHC arm, a majority of women preferred to receive their results by text (34%) or cell phone call (36%). In the health facility arm, most women preferred to return to the health facility to obtain their self-test results (62%).

**TABLE 2:**
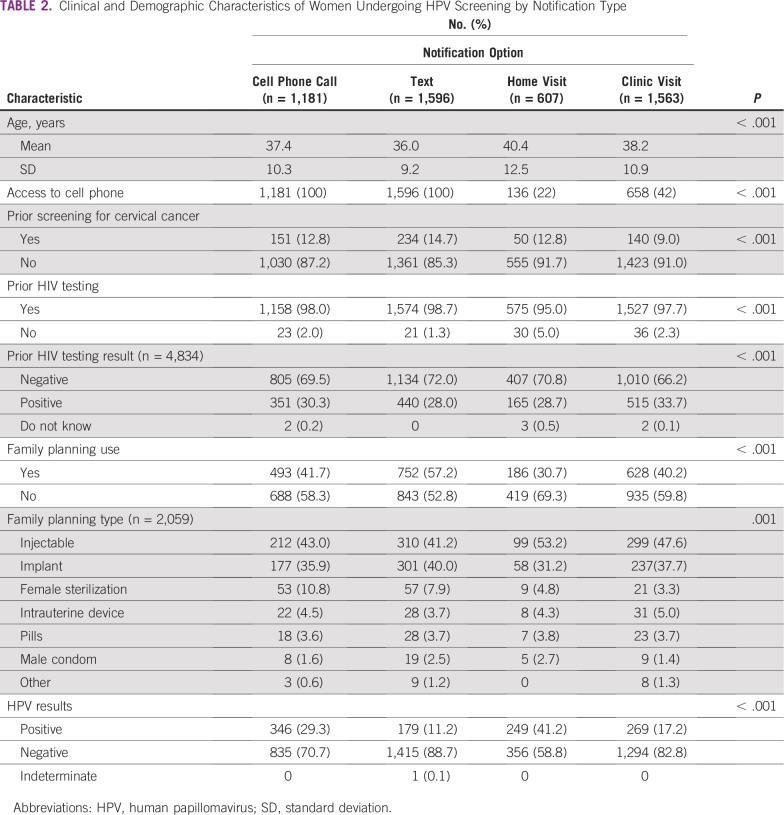
Clinical and Demographic Characteristics of Women Undergoing HPV Screening by Notification Type

Text messaging was the most successful route for notification of results, with 100% confirmation that the texts were received (Table 3). This was followed by home visit (98%), cell phone call (83%), and return clinic visit (62%). Among 84 HPV-positive women who did not return to the clinic for their results, 11 received their results through a home visit. Route of notification was significantly associated with treatment access. Almost half of women who received their results at a home visit (45%) accessed treatment, compared with women who opted for text (38%), cell phone call (38%), or clinic visit (23%; *P* < .001). In the subset of 196 women who opted for a return clinic visit and actually received their results, 62 (32%) successfully accessed treatment. On multivariable analysis controlling for prior screening, HIV testing, and contraceptive use, only clinic visits remained significantly associated with decreased odds of treatment (adjusted odds ratio, 0.45; 95% CI, 0.29 to 0.70) and time to treatment compared with text messaging.

**TABLE 3:**
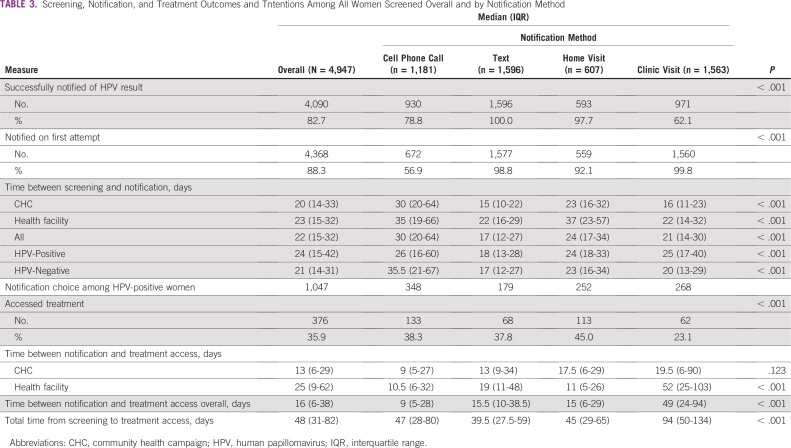
Screening, Notification, and Treatment Outcomes and Tntentions Among All Women Screened Overall and by Notification Method

There was a statistically significant difference in time between screening and receipt of results by notification method, with text messaging being the most efficient strategy (17 days; IQR, 12-27). The same pattern was also seen in time between screening and treatment. HPV-positive women who received their results through a return clinic visit had the longest median delay between screening, notification, and treatment at 49 days (IQR, 24-94 days), compared with 9 days (IQR, 5-28 days) for cell phone calls, 15.5 days (IQR, 10-38.5 days) for texts, and 15 days (IQR, 6-29 days) for home visits (*P* < .001). There were significant differences in median time between screening and notification by study arm (CHC arm, 20 days *v* health facility arm, 23 days; *P* = .004) and between notification and treatment access among HPV-positive women (CHC arm, 13 days *v* health facility arm, 25 days; *P* < .001). These differences went away when controlling for notification preferences.

## DISCUSSION

This study showed that women were able to receive and act upon HPV results provided to them by cell phone, either through a call or text message. Despite the fact that text messaging was the most efficient way to receive results, it was not associated with higher rates of treatment access. Overall, returning to the clinic to obtain results was an inefficient and ineffective strategy, requiring a second visit for participants, reaching fewer than two thirds of women tested and resulting in lower treatment uptake for those who needed it. Given the overall low rate of treatment acquisition, notification strategies need to be addressed, along with other significant treatment barriers, such as distance to treatment center, transportation costs, and health understanding, to make this two-step screen-and-treat program more effective.

Although mHealth strategies are promising ways to address many of the gaps in service delivery in rural areas, efficacy is limited by lack of cell phone access in some areas. In this study, 72% of women had access to a cell phone they were comfortable using to receive results. This rate is also lower than what has been seen in other studies and reports.^18,19^ This difference may result from the rural and impoverished study setting, lower cell phone ownership among women compared with men, or unwillingness to receive potentially sensitive health information by cell phone.^20^ We also found that women who reported access to cell phones had other characteristics that suggested health-seeking behavior, such as family planning use and prior HIV testing. The low rates of cell phone access among women who opted for home visits or clinic pick-up suggests that one of the driving factors in choosing a cell phone–based method was simply owning or having access to a cell phone. However, the fact that more than 40% of the women opting to collect their results at the clinic had a cell phone suggests acceptability, privacy, and possibly literacy were barriers.

The difference in choice of notification method had real clinical consequences in this study. Opting to return to the clinic for results was the least successful strategy, resulting in a low proportion of women successfully receiving results (62%) and an even lower proportion accessing treatment (23%). Although text messaging and cell phone calls were the most efficient notification methods, there were lower rates of treatment acquisition in these groups than among women who received their results via home visit. The lower rates of cell phone ownership or access in the home visit group would potentially suggest more difficulty in navigating treatment, so it seems that the opportunity to answer questions, along with the time, personal counseling, and encouragement for follow-up inherent in a home visit, was a key factor in encouraging treatment access. In addition, home visits were carried out by CHVs from the participants’ communities, likely people with whom they had a pre-existing relationship and whom they could possibly trust in their health care advice. An optimal notification strategy would combine the health information and support of a one-on-one visit from a reliable source with the efficiency and privacy of the cell phone–based notification strategies. This may be successful in the form of enhanced scripts for cell phone calls, increased face-to-face time with CHVs at the time of screening, or improvement in counseling materials provided to patients after testing.

This study was unique in that it used a robust design to compare implementation processes and strategies for HPV testing. This allowed us to look at the factors that made women choose their notification strategy, whereas a randomized trial of notification strategies would have, by necessity, eliminated a large number of the participants who could not or would not choose a cell phone–based notification method. Because these are often the most hard-to-reach women, we felt that a study design that eliminated them would not address the overall study question of how to increase access to effective screening. However, given the chosen study design, we do not want to overstate the association between method of results notification and access of treatment, given the myriad confounding factors that contribute to the selection of that method.

An additional limitation is the lack of specific information on notification via either text message or clinic visit. Although text message receipt was confirmed, there was no way to ensure that the participant herself had read the message. Regarding clinic pick-up, missing data entry for the dates on which the results were collected from clinic led us to use the date on which results were provided to the clinic as the notification date. This was the most conservative strategy, because it underestimated the time to notification, biasing a comparison with other methods (which all had faster turnaround) toward the null hypothesis of no difference. We are therefore confident that the observed statistical differences are real, even if the actual magnitude of the difference is greater.

Cell phones and other mHealth interventions have the potential to bridge infrastructural and informational gaps in many settings. Among women in low-resource settings, these strategies may increase access to health information that had previously been controlled by male partners or had been inaccessible because of distance from health facilities or poor translation into understandable messaging. This study shows that women in a rural area of western Kenya with limited health care infrastructure were willing and able to use cell phones to receive the results from an HPV testing campaign. This may be an important addition to an HPV-based screening strategy to reduce the additional visit for results. This also opens the door to the possibility of the wider use of cell phones in cervical cancer prevention strategies and other reproductive health interventions. Although text messaging allows for more automation and efficiency, there are advantages to personalized messaging. The results also made it clear that there are other barriers to accessing the treatment necessary to make cervical cancer prevention programs effective and that cell phone coverage and comfort are not universal. mHealth is a promising strategy; however, any next steps must take into account the multiple behavioral and logistical barriers that ultimately affect a woman’s ability to access health care.

## References

[1] Bruni L, Barrionuevo-Rosas L, Albero G, et al: Human Papillomavirus and Related Diseases Report: Kenya. https://hpvcentre.net/statistics/reports/KEN.pdf

[2] Bosch FX Castellsagué X de Sanjosé S HPV and cervical cancer: Screening or vaccination? Br J Cancer 98 15 21 2008 18182975 10.1038/sj.bjc.6604146 PMC2359713

[3] World Health Organization: WHO Guidelines for Screening and Treatment of Precancerous Lesions for Cervical Cancer Prevention. https://apps.who.int/iris/bitstream/handle/10665/94830/9789241548694_eng.pdf?sequence=1 24716265

[4] Denny L Kuhn L Hu CC et al Human papillomavirus-based cervical cancer prevention: Long-term results of a randomized screening trial J Natl Cancer Inst 102 1557 1567 2010 20884893 10.1093/jnci/djq342

[5] Sankaranarayanan R Nene BM Shastri SS et al HPV screening for cervical cancer in rural India N Engl J Med 360 1385 1394 2009 19339719 10.1056/NEJMoa0808516

[6] Ogilvie GS Mitchell S Sekikubo M et al Results of a community-based cervical cancer screening pilot project using human papillomavirus self-sampling in Kampala, Uganda Int J Gynaecol Obstet 122 118 123 2013 23731506 10.1016/j.ijgo.2013.03.019

[7] Hampshire K Porter G Owusu SA et al Informal m-health: How are young people using mobile phones to bridge healthcare gaps in Sub-Saharan Africa? Soc Sci Med 142 90 99 2015 26298645 10.1016/j.socscimed.2015.07.033

[8] US Agency for International Development: mHealth Compendium, Volume 5. http://www.msh.org/sites/msh.org/files/_2015_08_msh_mhealth_compendium_volume5.pdf

[9] Jennings L Omoni A Akerele A et al Disparities in mobile phone access and maternal health service utilization in Nigeria: A population-based survey Int J Med Inform 84 341 348 2015 25737460 10.1016/j.ijmedinf.2015.01.016

[10] GSMA: Striving and Surviving: Exploring the Lives of Women at the Base of the Pyramid. https://www.gsma.com/mobilefordevelopment/wp-content/uploads/2013/01/GSMA_mWomen_Striving_and_Surviving-Exploring_the_Lives_of_BOP_Women.pdf

[11] Jennings L Gagliardi L Influence of mHealth interventions on gender relations in developing countries: A systematic literature review Int J Equity Health 12 85 2013 24131553 10.1186/1475-9276-12-85 PMC4015705

[12] Kenya National Bureau of Statistics: Kenya Demographic and Health Survey 2008-09. https://dhsprogram.com/pubs/pdf/fr229/fr229.pdf

[13] World Health Organization: mHealth: New Horizons for Health Through Mobile Technologies: Second Global Survey on eHealth. https://www.who.int/goe/publications/goe_mhealth_web.pdf

[14] Odeny TA Bailey RC Bukusi EA et al Text messaging to improve attendance at post-operative clinic visits after adult male circumcision for HIV prevention: A randomized controlled trial PLoS One 7 e43832 2012 22957034 10.1371/journal.pone.0043832 PMC3434192

[15] Odeny TA Bailey RC Bukusi EA et al Effect of text messaging to deter early resumption of sexual activity after male circumcision for HIV prevention: a randomized controlled trial J Acquir Immune Defic Syndr 65 e50 e57 2014 23846561 10.1097/QAI.0b013e3182a0a050 PMC3867588

[16] Odeny TA Bukusi EA Cohen CR et al Texting improves testing: A randomized trial of two-way SMS to increase postpartum prevention of mother-to-child transmission retention and infant HIV testing AIDS 28 2307 2312 2014 25313586 10.1097/QAD.0000000000000409 PMC4834137

[17] Huchko MJ, Ibrahim S, Blat C, et al: Cervical cancer screening through human papillomavirus testing in community health campaigns versus health facilities in rural western Kenya. Int J Gynaecol Obstet 141:63-69, 2018 10.1002/ijgo.12415 PMC7369666 29197067

[18] World Health Organization: WHO-CHOICE. https://www.who.int/choice/cost-effectiveness/en/

[19] International Telecommunications Union: World Telecommunication/ICT Development Report 2010: Monitoring the WSIS Targets—A Mid-Term Review. https://www.itu.int/dms_pub/itu-d/opb/ind/D-IND-WTDR-2010-PDF-E.pdf

[20] Crandall A, Otieno A, Mutuku L, et al: Mobile phone usage at the Kenyan base of the pyramid. http://documents.worldbank.org/curated/en/187531468048273017/Mobile-usage-at-the-base-of-the-pyramid-in-Kenya

